# Whole Genome Sequencing Analysis of Porcine Faecal Commensal *Escherichia coli* Carrying Class 1 Integrons from Sows and Their Offspring

**DOI:** 10.3390/microorganisms8060843

**Published:** 2020-06-04

**Authors:** Tiziana Zingali, Cameron J. Reid, Toni A. Chapman, Daniela Gaio, Michael Liu, Aaron E. Darling, Steven P. Djordjevic

**Affiliations:** 1 The ithree institute, University of Technology Sydney, Sydney NSW 2007, Australia; tiziana.zingali@student.uts.edu.au (T.Z.); cameron.reid@uts.edu.au (C.J.R.); daniela.gaio@student.uts.edu.au (D.G.); michael.liu@uts.edu.au (M.L.); aaron.darling@uts.edu.au (A.E.D.); 2 NSW Department of Primary Industries, Elizabeth MacArthur Agricultural Institute, Menangle NSW 2568, Australia; toni.chapman@dpi.nsw.gov.au

**Keywords:** porcine *E. coli*, commensal *E. coli*, class 1 integron, microbial genomic epidemiology, antimicrobial resistance

## Abstract

Intensive pig production systems often rely on the use of antimicrobials and heavy metal feed additives to maintain animal health and welfare. To gain insight into the carriage of antimicrobial resistance genes (ARGs) in the faecal flora of commercially reared healthy swine, we characterised the genome sequences of 117 porcine commensal *E. coli* that carried the class 1 integrase gene (*intI1*^+^). Isolates were sourced from 42 healthy sows and 126 of their offspring from a commercial breeding operation in Australia in 2017. *intI1*^+^ *E. coli* was detected in 28/42 (67%) sows and 90/126 (71%) piglets. Phylogroup A, particularly clonal complex 10, and phylogroup B1 featured prominently in the study collection. ST10, ST20, ST48 and ST361 were the dominant sequence types. Notably, 113/117 isolates (96%) carried three or more ARGs. Genes encoding resistance to β-lactams, aminoglycosides, trimethoprim, sulphonamides, tetracyclines and heavy metals were dominant. ARGs encoding resistance to last-line agents, such as carbapenems and third generation cephalosporins, were not detected. IS*26*, an insertion sequence noted for its ability to capture and mobilise ARGs, was present in 108/117 (92%) *intI1*^+^ isolates, and it played a role in determining class 1 integron structure. Our data shows that healthy Australian pig faeces are an important reservoir of multidrug resistant *E. coli* that carry genes encoding resistance to multiple first-generation antibiotics and virulence-associated genes.

## 1. Introduction

*Escherichia coli* (*E. coli*) comprises an important component of the commensal gastrointestinal flora of warm-blooded vertebrate species. Its evolution is characterised by a remarkable capacity to acquire mobile genetic elements (MGE) such as plasmids, genomic islands and phages by lateral gene transfer (LGT), which is the major driver of antimicrobial resistance (AMR) and pathogen evolution [1]. Antibiotic usage features prominently in efforts to curtail the impact of the pathogenic *E. coli* disease in livestock production systems and prevent animal loss. In addition to antibiotics, diets designed to enhance growth performance and pig health often contain zinc and copper [2,3], which influence the composition of the intestinal microbiota and are the major non-antibiotic growth promoters used in swine production [4,5]. Due to the rising global demand for animal protein, antimicrobial usage is forecast to increase substantially over the next 15 years [6]. In regards to pig production, more than 1.4 billion pigs are slaughtered globally on an annual basis for food consumption [7], a production process that generates billions of tonnes of faecal waste, where *E. coli* is present at 10^4^–10^8^ per gram of faeces [8]. The application of manure as land fertiliser introduces faecal bacteria, antimicrobial and heavy metal residues into the environment beyond the farm [9,10,11]. The impact of composting on the carriage of multidrug resistant (MDR) bacteria is complex, and recent evidence suggests that the practice is not effective in reducing antimicrobial resistance gene (ARG) pollution in animal manure [11]. Although the precise ecological and molecular effects of livestock manure as a land fertiliser are poorly understood and undoubtedly complex [11,12,13], this practice may represent the driving force for AMR spread in the environment, wildlife and humans [9,14].

Class 1 integrons are considered a reliable predictor of AMR as they play a crucial role in the capture and expression of gene cassettes encoding resistance to a wide variety of antimicrobials that are embedded in complex resistance regions associated with insertion sequences (IS) [15,16]. It has been estimated that the faecal production of humans and livestock is responsible for the release of 10^23^ copies of clinical class 1 integrons into the environment daily [17], and large-scale molecular screening studies have reported the contamination of estuary sediments and animal manure with class 1 integrons [13,17,18,19]. Therefore, understanding the extent of class 1 integron presence in intensive farming systems becomes crucial to containing the impact of AMR spread in animal, human and environmental settings. IS elements play a pivotal role in the capture and mobilisation of ARGs. IS*26*, in particular, pervades the genomes of a wide variety of *Enterobacteriaceae* [20,21,22,23] and is prevalent in commensal porcine *E. coli* residing on diverse plasmid backbones [21,24,25]. IS*26* is responsible for the mobilisation of a wide array of ARGs conferring resistance to first generation antimicrobials [25,26] and last-line agents such as third generation cephalosporins and colistin [27,28,29], and it is capable of shaping the structure of MGEs [30]. Therefore, we can expect MDR *Enterobacteriaceae* to continue evolving by capturing different combinations of ARGs with the potential to create new MDR pathogenic clones [31,32].

Sows are actively involved in the acquisition of multiple drug resistance by newborn piglets. Previous studies investigated the use of antimicrobials in sows and the presence of AMR in the faecal *E. coli* of their offspring, showing an association between MDR *E. coli* found in the two animal groups that persisted to slaughter [33,34]. These studies underpin the significant role of sows as potential sources of MDR *E. coli* for the intestinal microbiota of newborn piglets [34,35]. Farming practices also play a role in the acquisition and spread of AMR. Extended spectrum beta-lactamase (ESBL) producing *E. coli* sourced from pigs and individuals working on farms shared pulsed field gel electrophoresis (PFGE) profiles, multi-locus sequence types (STs), ARGs and plasmid incompatibility types, suggesting transmission between pigs and humans [36]. Understanding transmission pathways between sows, newborn piglets and farmers is critical to avoiding the spread of MDR *E. coli* within farms and, more broadly, the environment.

Previously, we used whole genome sequencing (WGS) to characterise 103 class 1 integron-positive (*intI1*^+^) *E. coli*, isolated in 2007 from swine faeces from two commercial production facilities in New South Wales, Australia, with a history of antimicrobial use. Our study showed that *E. coli* belonging to commensal phylogroups A and B1 carried ARGs and diverse virulence-associated genes (VAGs) [25]. Here, we used WGS to further investigate the genomic features of 117 commensal *intI1*^+^ *E. coli* sourced from a commercial breeding operation in 2017. We estimated the carriage rates of class 1 integrase gene *intI1* and determined the class 1 integron structures carried by *E. coli* from the faeces of 42 healthy sows and 126 of their post-weaned piglets. We also characterised the carriage of ARGs and VAGs, STs, serotypes and phylogenetic associations. This study provides further baseline knowledge about the genomic features of MDR commensal *E. coli* sourced from healthy Australian pigs.

## 2. Materials and Methods

### 2.1. Field Animal Trial

Rectal faecal samples from 126 one-day post-weaned pigs sourced from a commercial pig breeding operation in Australia were collected in January 2017 at the Elizabeth MacArthur Agricultural Institute (EMAI), Menangle, NSW. Rectal faecal samples from 42 healthy sows, which gave birth to the 126 piglets, were also collected from the breeding operation and were dispatched to the EMAI microbiology laboratory for processing. Faecal samples were labelled and immediately inoculated in EC-broth for the culture of *E. coli.* Faeces were stored at −80 °C. At the time of sampling, the sows were not receiving antimicrobial treatment, whereas the piglets had never received antimicrobials before sampling.

### 2.2. Faecal Sample Collection, E. coli Isolation and Identification

Three grams of faecal material from each animal were homogenised in 15 mL of phosphate-buffered saline (PBS) solution using a Stomacher (Bio-Rad) for 1 min. To isolate *E. coli* colonies, 500 µL of homogenised faecal material was incubated at 37 °C overnight in EC enrichment broth (BD) and 50 µL was used to inoculate petri dishes containing MacConkey agar (BD). Three presumptive *E. coli* colonies were picked for each faecal sample. To confirm the identity of the typical dark pink lactose-fermenting colonies, crude boiled cell lysates were prepared, and an aliquot was used as template for a multiplex PCR containing primers spanning the *E. coli*-specific *uspA* gene [37] and the class 1 integrase *intI1* [38,39]. The cycling conditions for the multiplex PCR were as follows: 94 °C for 2 min (initial denaturation) followed by 30 cycles 94 °C for 20 s (denaturation), 60 °C for 20 s (annealing) and 72 °C for 30 s (polymerisation). The final extension cycle was performed at 72 °C for 5 min. The presence of *uspA* was sufficient to confirm the bacterial colony identity as *E. coli,* and *intI1* was considered as a reliable proxy for the presence of ARGs [40]. For sows’ faecal samples that were *intI1*^-^ after the first PCR screening, a further 10 dark pink lactose-fermenting colonies from the overnight enrichment broth were screened with the multiplex PCR.

### 2.3. Isolate Identifiers

*E. coli* isolates were identified with the TZ prefix. *E. coli* derived from sows were identified by TZ followed by the animal number and S (i.e., TZ2_S). *E. coli* from piglets were identified by the TZ number, which refers to the sow that gave birth to it, and the piglet number followed by P (i.e., TZ2_1P). To investigate the diversity of *E. coli* in a single faecal enrichment broth, we sequenced the genomes of all three *E. coli* colonies for two sows and six piglets. These isolates are indicated by the same code for the first bacterial colony, plus the suffix *a* or *b* for the second and third colonies (i.e., TZ17_4P*a*).

### 2.4. DNA Extraction and Whole Genome Sequencing

Genomic DNA was extracted using the DNeasy Blood and Tissue Kit (Qiagen), following the manufacturer’s standard protocol for bacterial cells. The steps used for DNA quantification and library preparation for WGS were performed as described previously [25]. After multiplex PCR screening, 117 *intI1*^+^ *E. coli* genomes (32 from sows and 85 from piglets) were sequenced using an Illumina HiSeq 2500 v4 instrument. The Illumina raw reads were assessed for quality and assembled using the A5 pipeline (version A5-miseq 20150522) [41]. For eight animals (two sows and six piglets) we selected three *intI1^+^ E. coli* colonies to be sequenced to determine if only a single *intI1*^+^ *E. coli* clone was dominating per faecal sample. At least two colonies were successfully sequenced for 7 out of 8 pigs.

### 2.5. Assembly Statistics

Assembly statistics for 117 sequenced *intI1^+^ E. coli* are provided in Table S1. The number of scaffolds per genome ranged from 100 to 864, with an average of 247. The median sequencing coverage was between 28× and 94×, with an average of 58×.

### 2.6. Gene Identification, Serotyping, Phylogrouping, Phylogenetic Analysis and Multilocus Sequence Typing (MLST)

Antimicrobial resistance and virulence genes were catalogued using local BLAST v2.2.30+ and the previously reported gene databases [25] (Tables S2–S4). These were also used to identify plasmid incompatibility groups, insertion sequences and serotypes, addressing the same criteria of >90% nucleotide sequence identity [25]. Identification of truncated gene sequences, class 1 integron characterisation and confirmation of the presence of two integrons in the collection was performed as previously described [25].

*E. coli* phylogroups and MLST determination were performed in silico using the Clermont et al. scheme [42] and the PubMLST database (http://pubmlst.org/) following the scheme proposed by Achtman (http://mlst.warwick.ac.uk/mlst/).

The short read alignment of our strains to K12-MG1655 (ST10) as a reference genome was performed as previously described using the custom snakemake workflow snplord, available at https://github.com/CJREID/snplord. The full alignment was recombination filtered and a tree inferred by Gubbins using RaxML as a tree-builder. This tree and recombination-filtered alignment are available via Figshare (See data availability for links). Tree analysis and visualisation with metadata was performed with iTOL (https://itol.embl.de).

### 2.7. Ethical Statement

The swine trial was conducted at the Elizabeth Macarthur Agricultural Institute under the Animal Ethics Committee number M16/04.

### 2.8. Data Availability

The following data arising from this study have been made publicly available:

1. One hundred and seventeen short read pairs and corresponding draft genome assemblies of *E. coli* as described in this project were deposited in GenBank under BioProject PRJNA509690. Individual sample accession numbers can be found in Table S1. https://www.ncbi.nlm.nih.gov/bioproject/PRJNA509690
2. Maximum likelihood phylogenetic tree of 185 faecal *E. coli* whole genome sequences from Australian pigs. https://doi.org/10.6084/m9.figshare.12233744
3. Recombination filtered alignment of 185 faecal *E. coli* whole genome sequences from Australian pigs. Aligned to K12-MG1655 complete genome with Snippy and recombination filtered with Gubbins. https://doi.org/10.6084/m9.figshare.12233747

## 3. Results

### 3.1. Class 1 Integrase Gene Presence

We isolated three *E. coli* colonies per sample from a cohort of 42 healthy sows and 126 post-weaned piglets and screened them for the presence of the *intI1* gene. *intI1* was detected in 28/42 sows (67%) and 90/126 piglets (71%). For the 14 sows in which *intI1* was not detected in the initial screen, we tested a further 10 *E. coli* colonies. Using this approach, almost all sows (41/42; 98%) were found to carry *intI1*^+^ *E. coli* in their faeces.

### 3.2. Phylogroups, Sequence Types and Serotypes

The commensal *E. coli* from sows and their offspring had their phylogroup, MLST and serotype determined in silico. Phylogroup A was predominant (70/117; 60%), followed by phylogroups B1 (36/117; 31%), D (9/117; 7.6%) and B2 (2/117; 1.7%). Phylogroup A was also dominant in sows and piglets when considered separately. Interestingly, 9/12 (75%) *E. coli* isolates belonging to pathogenic phylogroups B2 and D were found in piglets. Phylogroup B1 *E. coli* was more prevalent in piglets than in sows.

Forty-six different STs were identified in the study collection, 19 of which were represented by a single isolate. ST10 was most common (10/117; 8.5%), followed by ST20 (8/117; 6.8%) and ST48 (7/117; 6%). ST29 and ST398 were also frequently encountered but only in piglets, while ST48 was identified in both groups but predominantly in piglets. Whilst 13 STs were identified in both sows and piglets, there was only one instance where a piglet shared an ST with its mother.

The in silico serotyping analysis identified 52 different serotypes among 103 isolates. Nine isolates were O non-typable (ONT) and associated with 7 different H alleles; one isolate typed as ONT:HNT. *E. coli* isolates with the same ST often displayed the same serotype, though ST10 in particular displayed a variety of serotypes. Serotypes O15:H2 (8/85; 9.4%) and O15:H11 (5/85; 5.9%) were predominant in piglets but absent in sows, while O8:H30 was prevalent in sows (4/32; 12.5%) and frequently encountered in piglets.

### 3.3. Phylogenetic Analysis

**Figure 1:**
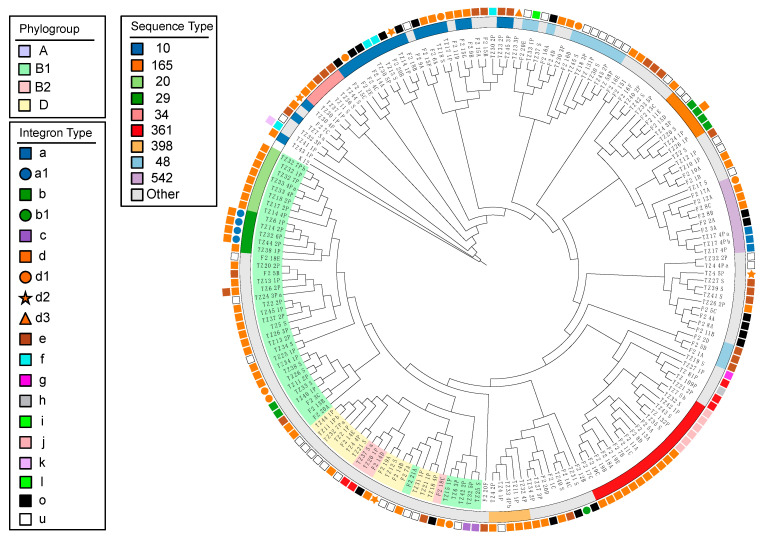
Maximum likelihood phylogeny shown as cladogram. The cladogram shows the 117 porcine commensal *E. coli* genomes from the present study as well as 68 from the second farm described in our previous study (labelled “F2”). Label highlight colour indicates phylogroups. Sequence types are represented by a coloured strip. Class 1 integron structures in Figure 3 are annotated with letters (a- l). “o” (black square) indicates that another integron not shown in Figure 3 was identified. “u” (white square) indicates that the integron structure was not determined.

In order to visualise genetic relatedness among our collection, we inferred a maximum likelihood phylogenetic tree from an alignment of our 117 sequences to the complete genome of K12-MG1655 (Figure 1). We also included a previously examined porcine commensal *E. coli* collection of 68 genomes from 2007 for comparison. These strains are labelled with the prefix “F2”.

The phylogeny was dominated by phylogroup A strains, separated from all other phylogroups on one major clade. The phylogroup A clade contained seven of the nine most common STs, five of which were shared between the 2017 and 2007 collections. Sequence types belonging to the ST10 complex were mostly clustered together, except for ST34, which split two clusters of ST10 and a cluster of three ST48s that were distinct from the other ST48s in the collection.

The second major clade comprised strains from all four phylogroups but mostly featured phylogroup D, including two ST117 strains, one from each collection. The third major clade was made up of B1 and a singular A strain. This clade contained ST20 and ST29, common STs observed in the 2017 collection only. There were numerous instances of strains from the two collections clustering together in sub-clades within all three of the major clades, particularly phylogroup A.

### 3.4. Antimicrobial Resistance Genes (ARGs)

In total, 21 different ARGs were identified within the collection, and each isolate carried between two and 17 ARGs. 113/117 *intI1*^+^ strains (96.6%) carried ARGs conferring resistance to one antimicrobial in three or more categories and were therefore classified as MDR [43].

**Figure 2:**
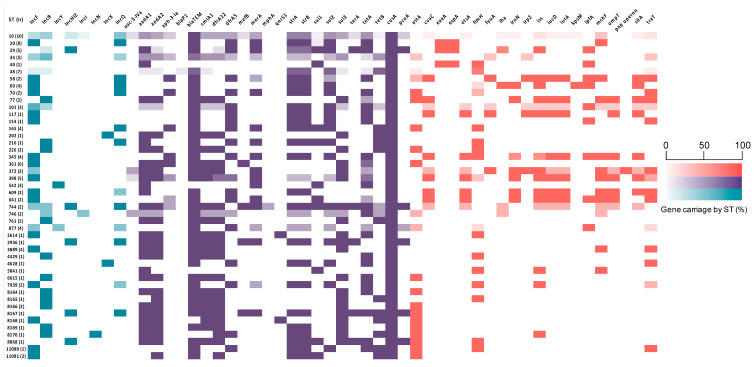
Heat map showing proportion of sequence types (STs) carrying plasmid replicons (aqua), antimicrobial resistance genes (purple) and virulence-associated genes (red). For the full dataset see Supplementary Tables S2–S4.

**Figure 3:**
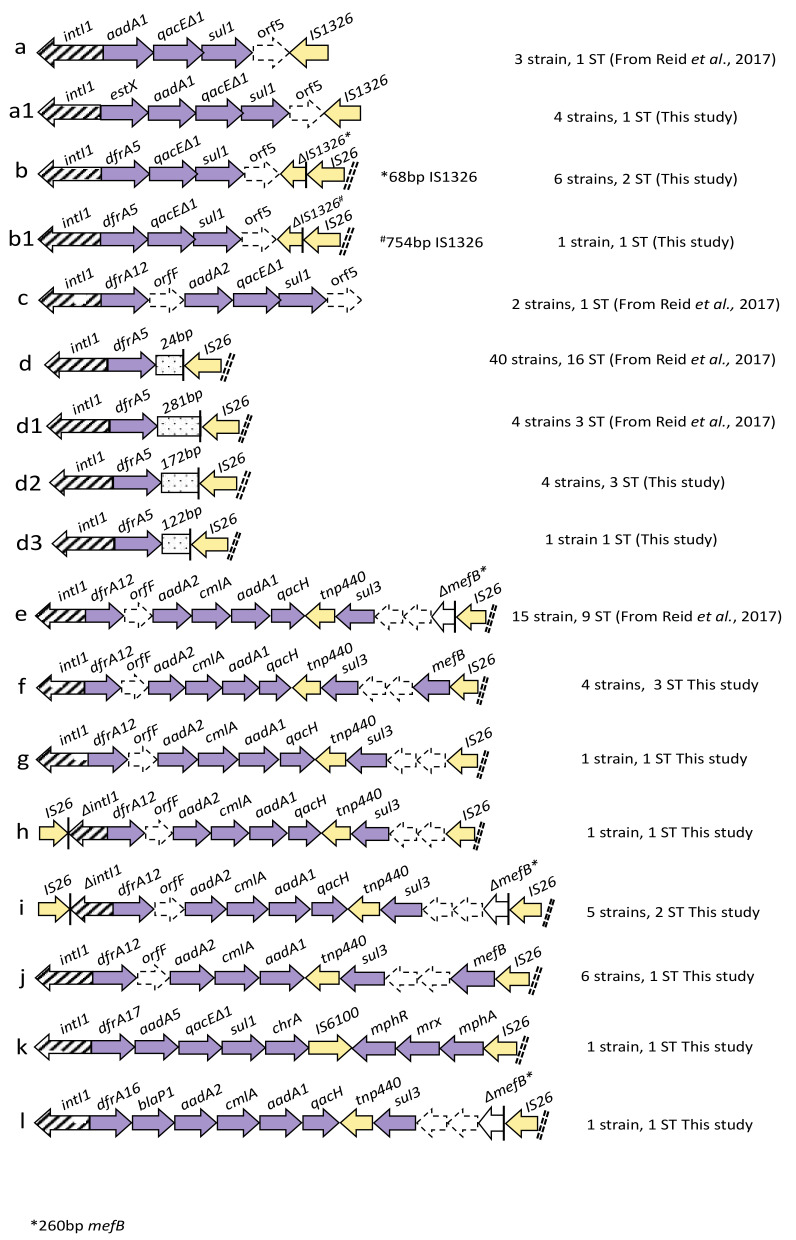
Schematic representation of class 1 integron structures (labeled a-l) present in the sequenced genomes. Arrows represent open reading frames (ORFs). Purple and yellow arrows represent antimicrobial resistance genes (ARGs) and insertion sequences, respectively. Arrows with dashed lines indicate hypothetical proteins. Diagonal dashed double lines represent scaffold breaks in the sequence.

The predominant ARGs identified in the collection were *bla*_TEM-1_ (108/117; 92%), followed by *strA*, *strB* (69/117; 59%) and *tetA* (65/117; 55.5%) (Figure 2). Frequently observed resistance gene cassettes associated with class 1 integrons included *dfrA5* (56/117; 48%), *dfrA12* (102/117; 87%), *aadA1* (63/117; 54%), *aadA2* (53/117; 45%) and *cmlA* (49/117; 42%) encoding trimethoprim, aminoglycoside and chloramphenicol resistance, respectively (Figure 3). *aph(3`)-Ia*, conferring resistance to neomycin and kanamycin, was present in 28/117 isolates (24%). Three sulphonamide-resistance genes were identified in the collection. *sul2* was predominant (60/117; 51%), followed by *sul3* (49/117; 42%) and *sul1* (24/117; 20.5%). *sul2* also predominated in piglets (50/85; 59%), followed by *sul3* (30/85; 35.2%) and *sul1* (20/85; 23.5%). Contrarily, in sows, *sul3* was most prevalent (19/32; 59.3%), followed by *sul2* (10/32; 31.2%) and *sul1* (4/32; 12.5%). The *sul1* and *sul3* genes were found to be closely associated with class 1 integrons; however, integrons lacking *sul1* due to IS*26* were also a feature of the collection (Figure 3). Considering the animal groups singularly, bla_TEM-1_ was prevalent in both, followed by *dfrA12*, *aadA1* and *aadA2* in sows and *strA, strB, sul2* and *tetA* in piglets. Except for the single ARGs identified only in piglets, such as *qnrS, blaP1* and *mphA*, all the ARGs that were identified in the study collection were present in both piglets and sows.

Genes encoding resistance to third generation cephalosporins, carbapenems and polymyxins were not detected. Resistance genes conferring resistance to mercury and tellurite were detected in 102/117 (87%) and 24/117 (20.5%) isolates, respectively. Chromosomally-encoded copper resistance genes (*cusA, cueO, copA*) were detected in 100% of the isolates, whilst plasmid-encoded genes such as *pcoA* were detected in 3/32 (9.4%) isolates from sows and 12/85 (14.1%) isolates from piglets (Figure 2, *cueO, copA* not shown).

### 3.5. Class 1 Integron Structures

We identified 17 different class 1 integron structures in this study. Six integrons (Figure 3a–c,k) carried *sul1*. The difference between structures (a) and (a1) was an additional gene cassette (*estX*), whereas structure (b1) represented a variant of (b) for the presence of two different truncation events, presumably mediated by IS*26* (Figure 3b,b1). Integron structure (d), with a truncated 3′-CS region, was identified in 40 isolates (40/117; 34%) and associated with 16 different STs (Figure 3d). Two novel IS*26*-mediated truncations of the 3′-CS region, one comprising 172 nucleotides and the other 122 nucleotides, respectively, were also identified (Figure 3(d2,d3)). A commonly identified *sul3*-associated integron comprised *dfrA12-orfF-aadA2-cmlA-aadA1-qacH-tnp440-sul3* (Figure 3e), and variants of this structure were also characterised. Integron (e) was the second most frequently reported structure in the collection. The length of the macrolide efflux gene *mefB* was one of the features that differentiated *sul3*-associated integrons with similar gene cassette arrays Figure 3(e–j). Structure (f) carried a full copy of *mefB* along with (j), which was missing the *qacH* gene cassette. Structures (e) and (i) carried a signature *mefB* 260 bp deletion (*ΔmefB*_260_), whereas (g) and (h) lacked *mefB*. In all the aforementioned structures, *mefB* was flanked by IS*26*, the presumptive cause of these truncation and deletion events.

Seven isolates carried two integron structures: (a1, d) 4/7; (d, e) 1/7; (b, d) 1/7; (f, k) 1/7. Class 1 integron structures (d) and (e) were the most prevalent in both groups, followed by (j) in sows and (i) in piglets. Nine integron types, (b), (c), (d), (d1), (d2), (e), (f), (i) and (j), were shared by sows and piglets. Integron structures (k) and (l) were each identified only once, both times in piglets. At least one representative for each integron structure was found in piglets, whereas in sows, structures (a), (a1), (h), (k) and (l) were not reported.

The mapping integron carriage data to the phylogeny (Figure 1, Tables S2 and S6) highlighted the carriage of certain integrons within multiple unrelated strains. Integrons (b), (d), (d1), (d2), (e), (f) and (i) were each present in more than one ST. Integrons (d), (d1) and (e) were also present in multiple STs within the 2007 collection.

### 3.6. Virulence Associated Genes (VAGs)

The most frequently identified VAGs in the collection were *fimH* (31/117; 26.4%), a type 1 fimbrial adhesin involved in bladder colonisation [44,45]; *iss* (36/117; 31%), the increased serum survival protein widely distributed in avian and human *E. coli* [46]; and *traT* (30/117; 26%), an outer membrane serum survival-associated protein. Less common VAGs in the collection were *eaeA* and *espA*, which are involved in the formation of the attaching and effacing lesions induced by enteropathogenic *E. coli* (EPEC) [47,48], ColV genes (*cvaA, cvaB*) and siderophore encoding genes (*iucD, iutA, irp2, fyuA and iroN*). Two isolates from phylogroup B2 carried the pap operon encoding for P fimbriae, which is one of the most recognised extra-intestinal virulence factors [49].

Within phylogroup A, 16/20 different VAGs were identified, with 18/20 in phylogroup B1, 13/20 in phylogroup B2 and 17/20 in phylogroup D. The highest number of VAGs per isolate was 12 for a single B2 isolate. Nonetheless, four phylogroup B1 and one phylogroup A isolates carried 11 VAGs each. Overall, only 15 phylogroup A isolates did not carry VAGs.

Heat-stable cytotoxin *astA* and *fimH* were the most common VAGs in sows, whereas *iss* and *mchF* (microcin H47), previously associated with extra-intestinal pathogenic *E. coli* (ExPEC) [50], were prominent in piglets. The membrane protein *traT* was the third most represented VAG in both groups.

### 3.7. Plasmid Incompatibility Groups

The collection was screened for eight plasmid replication-associated genes and all were represented in the collection (Figure 2). IncF was the most frequently identified (60/117; 51.2%), followed by IncR (27/117; 23%) and IncHI2 (12/117; 10.2%). All identified replicons except IncN were present in both sows and piglets.

## 4. Discussion

*E. coli* genomic sequences from the gastrointestinal tracts of healthy Australian pigs are underrepresented in public repositories. We recently described the genomes of 103 *intI1*^+^ *E. coli*, isolated from the faeces of mostly healthy swine that were sourced from two commercial production facilities with a history of antimicrobial usage [25]. Our previous study showed that commensal *E. coli* belonged predominantly to phylogroup A and B1 and sequence types ST10, ST361 and ST542. The present study provides further genomic data characterising 117 *intI1^+^* faecal commensal *E. coli* from healthy Australian sows and their offspring, sourced from a commercial breeding operation in 2017. The post-weaned pigs had not previously been exposed to antimicrobial treatment.

*E. coli* ST10 and ST20, belonging to phylogroup A and B1, respectively, were predominant in this study collection, a result that is consistent with earlier Australian studies [25,51]. ST10, part of clonal complex 10 (CC10), has been previously reported as an important extra-intestinal pathogen in humans and pigs [52,53] and a potential dominant clonal group of commensal *E. coli* globally [54]. The latter study also reported *E. coli* CC10 as well established in the gut of Australian pigs, belonging to multiple phylogenetic lineages [54]. A phylogenomic comparison of our 117 strains with a historical collection of *intI1^+^* commensal porcine *E. coli* from 2007 (Figure 1) revealed that, in addition to the conservation of STs, sub-lineages within these STs persisted in Australian swine. This suggests that *intI1^+^* CC10 *E. coli* remain under selection in Australian swine production systems, though the underlying mechanisms require further elucidation. Direct or indirect selection of antimicrobial resistance, and the genetic basis of commensal fitness, are avenues worth exploring. In addition, the gut microbiome of post-weaned piglets must quickly adapt to the transition from sow’s milk to a solid grain diet; during this period of approximately 10 days, animals are particularly prone to diarrhoeic disease [55,56]. Given the lack of diarrhoeal disease in the cohort and the presence of dominant clonal groups of commensal *E. coli* such as CC10, it is likely that these lineages are adept at surviving the transition phase and may have some protective effect from the risk of diarrhoeal disease. Therefore, further WGS of commensal *E. coli* CC10 is desirable in order to understand its intestinal persistence and any effect on the occurrence of post-weaning diarrhoea in piglets. *E. coli* ST20 was the second most represented ST in the collection, although it was only identified in the piglets’ group and not present in our historical collection. ST20 may represent an emerging ST carrying ARGs and VAGs in commensal porcine *E. coli* in Australia; however, further genomic studies are required to determine the extent of ST20 carriage in Australian porcine *E. coli* populations.

A wide variety of STs and serotypes were reported in the study collection, particularly in piglets. Previous studies evaluated the genomic diversity of intestinal *E. coli* in nursery pigs in the absence of antimicrobial treatment, suggesting that piglets showed significant genetic variety among *E. coli* [8]. To get an insight into *E. coli* diversity, we isolated and sequenced three *intI1*^+^ *E. coli* from a single porcine faecal sample for two sows and six piglets. Due to sequence failure episodes and the low number of colonies that were sequenced, we were not able to comment on the establishment of a single *E. coli* lineage or otherwise. Studies from Danish farms described the genetic relationship among *E. coli* sourced from pigs which had not previously been treated with antimicrobials [57,58]. These studies determined that sampling 10 *E. coli* colonies was sufficient to gauge *E. coli* diversity in the faeces of swine [57]. Moreover, much remains to be learned about the role of diet and animal age in the composition and concomitant carriage of AMR genes in the gut microbiota [33,34,59]. Collectively, our data suggest that *intI1^+^* commensal *E. coli* display remarkable genetic diversity. Further WGS studies are needed to determine the full genetic diversity of *E. coli* in the porcine gastrointestinal tract.

Farming practices, feed additives and the antimicrobial treatment regime given to sows may have a significant influence on how their offspring acquire ARGs [33,35]. In the present study, post-weaned piglets were not exposed to antimicrobial treatments prior to sampling; however, the sows were likely to have been previously exposed to antimicrobials. This may have been a contributing factor to the shared carriage of identical ARGs and the presence of *intI1* in both groups [33,60]. Surprisingly, we only identified one example of a sow and its progeny carrying the same ST (ST48). These two ST48 strains displayed identical ARG profiles. The general trend of shared ARGs but not STs may be due to the presence of too few strains isolated from sows for comparison but may also reflect that genes are simply more mobile or transmissible than organisms. Further work examining the context of ARGs in pigs and sows and more comprehensive sampling is required to address this.

Although the assembly of complex MDR genomic regions using short read sequencing technology is challenging, we identified 17 class 1 integron structures in which the *sul2* and *sul3* genes were prevalent. In *E. coli* from piglets, integron structure (d) was frequently identified in strains that also carried *bla*_TEM_, *strA, strB* and *sul2*, which are typical components of the transposon Tn*6029* [26]. This result suggests that the Tn*6029* transposon was present in the study collection, as highlighted in our previous study [25]*,* particularly in piglets. The presence of *aph(3′)-Ia*, conferring resistance to neomycin and kanamycin in 28 isolates, a gene that is typically flanked by IS*26*, suggests that Tn*6026* may also be a feature of MDR commensal *E. coli* in Australian swine production systems. Further studies using long read sequencing are needed in order to confirm these assertions. Integron structure (d), in which only 24 bp of the 3′-CS remains intact, is a frequently described genetic signature in Australia [61]. It was previously reported in atypical bovine enteropathogenic *E. coli* (aEPEC) throughout NSW, from the faeces and urine of humans with haemorrhagic colitis and UTIs, respectively, and in *E. coli* ST58 causing urosepsis in humans [24,61,62]. Our analysis demonstrates that this signature remains common within and between clonal lineages in Australian swine, supporting both vertical and horizontal gene transfer as the mechanisms by which it persists. In the present study, we identified two new class 1 integron structures carrying unique deletions in their 3′-CS, presumably created by the action of IS*26* (integrons d2; d3). This result suggests that Tn*6029* and Tn*6026* may truncate the 3′-CS of class 1 integrons in different locations, creating novel genetic signatures. Further studies are required to confirm this.

*sul3-*associated class 1 integrons were prevalent here and in our earlier study [25]. Since its first description in pigs [63], *sul3* has been reported in Australia in commensal *E. coli* and *Salmonella enterica* from swine [25,51,64] and commensal and uropathogenic *E. coli* from humans [65,66], but it has been reported less frequently in avian pathogenic *E. coli* [67]. Based on these observations, livestock may play an important role in the spread of MGEs that carry *sul3* and other sulphonamide resistance genes in *Enterobacteriaceae*. *sul3-*associated class 1 integrons may be tracked identifying different IS*26*-mediated *mefB* deletions. In our previous study, we described evidence that IS*26* was shaping atypical class 1 integron structures and causing characteristic deletions in *mefB* [25]. In our current study, we identified all four previously described *sul3*-associated integrons. Integron (e) in particular was most prevalent and was found in both predominant and sporadically occurring STs. We also identified six new variants, several of which had complete copies of the *mefB* gene which had not been previously reported in Australia. The presence of a full copy of *mefB,* as well as different deletion or truncation events, may indicate that class 1 integrons are mobilised with different mechanisms involving different MGEs such as transposons or ISs. Furthermore, variants of *mefB* may represent useful genetic epidemiological markers.

The co-carriage of ARGs and heavy metal resistance genes in the collection is concerning, yet unsurprising given the known association between class 1 integrons, ARGs and mercury resistance transposons [68]. In food animals, ARGs and heavy metal resistance genes in widely disseminated plasmid types, such as the HI2 family, have been previously reported [69,70]. This is best explained by widespread in-feed copper and zinc supplementation in swine operations, which drives the persistence of AMR in the absence of direct antimicrobial selection pressure. A body of evidence justifies the phasing out of both antimicrobials and heavy metals in food animal production in favour of alternatives [71].

The high rate of VAGs identified in the present study was unexpected, particularly because *E. coli* belonging to phylogroups A and B1 are usually considered as low virulence, commensal strains [72]. VAGs identified in *E. coli* from sows and piglets were previously reported in multiple pathotypes of *E. coli* [47,48,49,73,74], and the carriage of multiple VAGs in commensal *E. coli* strains is consistent with our earlier study [25]. Reliance on antibiotics and metals to prevent outbreaks of infectious disease is likely to not only maintain high carriage levels of class 1 integrons in plasmids that also carry heavy metal resistance genes and IS*26* [25,74,75], but it may yield a more severe disease presentation by driving the evolution of MDR hybrid *E. coli* with virulence attributes derived from different pathovar designations. Indeed, Australia has already experienced one such episode in early 2000s when an MDR enterotoxigenic *E. coli* (ETEC) O157:H19-ST4245 (ST23 complex) that was phylogenetically more closely related to serogroup O78 avian pathogenic *E. coli* (APEC) and distant from enterohaemorrhagic *E. coli* (EHEC,) O157:H7, caused widespread mortalities in several swine production operations [76]. Similarly, plasmids described in *Salmonella enterica* serovar Typhimurium 1,4 [5] 12:i:- from Australian pigs in 2013 were shown to carry EHEC VAGs that had been described in *E. coli* sourced from cattle circa 1998, highlighting how these elements can be mobilised and how VAGs and AMR genes can be shared [75]. Previous studies identified both VAGs and ARGs in multiple conjugative plasmid types in *Enterobacteriaceae* [22,75,77]. Hybrid plasmids are known to be generated by the mechanism of action of IS*26*, leading to the creation of plasmids carrying combinations of VAGs and ARGs [22,78], conferring a high selective advantage to the host strain. *E. coli* sourced from sows and piglets carried multiple VAGs. Notably, most of the hypervirulent strains, belonging to phylogroups B2 and D, were sourced from piglets. The significant presence of VAGs in this animal group represents a high risk of the onset of post-weaning diarrhea [79], considering the temporary disequilibrium in the gut microflora because of diet transition from liquid to solid. Altogether, these observations suggest that *E. coli* in commercial swine production systems should be monitored, as “harmless” commensals may rapidly transition into drug resistant pathogens via the acquisition of hybrid virulence/resistance plasmids which are present in the porcine faecal microbiome.

Collectively, our data shows that despite the evidence for sound antimicrobial stewardship in Australia [51,80], class 1 integron structures are present and are evolving in Australian swine breeding operations. This is in part due to the infiltration of IS*26*, which is altering class 1 integron structures by creating complex regions of resistance and enhancing the opportunities for the formation of hybrid MDR resistance and virulence plasmids [75,81]. Australian antimicrobial stewardship practices, in conjunction with strict quarantine requirements, prohibiting the import of living animals or fresh pork for sale [82], appear to be effective in limiting the capture and carriage of ARGs to last-line antimicrobials, such as third generation cephalosporins and colistin, in porcine *E. coli*. This may have had an influence on the apparently stable presence of class 1 integron structures in commensal porcine *E. coli* compared to our earlier findings [25]. Nonetheless, effective strategies for improving on-farm biosecurity routines and farmers’ education need to be implemented in order to prevent the spread of AMR and disease outbreaks.

Our WGS study suggests that Australian commensal porcine *E. coli* mainly belong to commensal phylogroups A and B1 and are an important reservoir of ARGs encoding resistance to first generation antimicrobials. Notably, the *E. coli* characterised here carry atypical class 1 integron structures, particularly those associated with *sul3*, and have been altered in large part due to the mechanism of action of IS*26*. Commensal *E. coli* also carry numerous and diverse VAGs, including those reported in different *E. coli* pathotypes, suggesting that their zoonotic potential needs to be monitored. Further genomic studies are needed to understand the role played by the surrounding environment and the sows’ intestinal microflora on the acquisition of MDR *E. coli* in post-weaned piglets not previously exposed to antimicrobial treatments. Furthermore, our data suggest that the microbiological impact of the application of swine manure on agricultural land needs to be monitored more closely, and they underscore the importance of adopting a one health perspective in the study of pathogen evolution and MDR [40,68].

## Supplementary Materials

The following are available online at https://www.mdpi.com/2076-2607/8/6/843/s1, Tables S1–S4.
